# Rose Bengal Is a Precise Pharmacological Tool Triggering Chloroplast‐Driven Programmed Cell Death in Plants, Dependent on Calcium and Mitochondria, and Associated With Early Transcriptional Reprogramming

**DOI:** 10.1002/pld3.70110

**Published:** 2025-10-20

**Authors:** Yasmine Jnaid, Rory Burke, Inge De Clercq, Joanna Kacprzyk, Paul F. McCabe

**Affiliations:** ^1^ School of Biology and Environmental Science University College Dublin Dublin Ireland; ^2^ Department of Plant Biotechnology and Bioinformatics Ghent University Ghent Belgium; ^3^ VIB Center for Plant Systems Biology Ghent Belgium

**Keywords:** chloroplast, oxidative stress, programmed cell death, Rose Bengal, singlet oxygen, stress signaling

## Abstract

Programmed cell death (PCD) mediates plant development and environmental interactions. Photosynthesis‐derived singlet oxygen (^1^O_2_) is one of key reactive oxygen species (ROS) implicated in acclimation and PCD responses to environmental stress conditions. Using *Arabidopsis thaliana* cell suspension culture system, we characterized the PCD induced by Rose Bengal (RB), a photosensitizer generating ^1^O₂ upon light exposure. Obtained results reiterated that RB‐induced PCD is light and chloroplast dependent. Further, we demonstrate that PCD induced by RB involves calcium signaling and mitochondria, thus sharing common features with other forms of regulated cell death in plants. The PCD induced by RB was associated with early transcriptional reprogramming, involving switching off the primary metabolism and activation of stress response and cell death related pathways (e.g., oxidative stress, hypoxia, immunity, and salicylic acid). The constructed gene regulatory network featured ^1^O_2_‐responsive genes and suggested involvement of transcription factor *ANAC102* in retrograde regulation of RB‐induced PCD. Interestingly, treatment with RB also induced light independent toxicity, showing features of uncontrolled, necrotic cell death. Presented findings highlight RB as a valuable tool for studying ^1^O_2_‐induced PCD that may advance future work on chloroplast‐mediated oxidative stress responses and enhancing plant resilience to climate change‐related stressors through targeted modulation of ROS pathways.

## 1 Introduction

Programmed cell death (PCD) is a genetically encoded and tightly regulated form of cellular suicide (Ameisen 2002). In plants, PCD is controlled by multiple signaling pathways and plays an essential role in development and modulation of responses to biotic stimuli and abiotic stresses (Heath 2000; Olvera‐Carrillo et al. 2015; Daneva et al. 2016; Burke et al. 2020). There are numerous examples of PCD events being a normal part of vegetative and reproductive plant development, including differentiation of xylem, tapetum, and root cap cells (Kumpf and Nowack 2015; Xie et al. 2022); PCD induced by pollen self‐incompatibility (Wilkins et al. 2014); cell death related to tissue remodeling during aerenchyma formation and leaf morphogenesis (Gunawardena et al. 2001; Gunawardena et al. 2004); or PCD finalizing senescence (Rogers 2015). PCD can be both beneficial and detrimental in the context of biotic and abiotic interactions (Locato and De Gara 2018). Hypersensitive response (HR) restricts the growth of biotrophic pathogens (Heath 2000), PCD in the root stem cell niche promotes cold acclimation (Hong et al. 2017), and PCD triggered by the excess excitation energy stress activates systemic acclimation to high light (Wituszyńska and Karpinski 2013). However, high levels of PCD activated by severe abiotic stress are detrimental to plant health and survival, as is cell death activated by necrotrophic pathogens (Coll et al. 2011). Understanding of plant PCD and its regulation at a cellular and organism level is therefore required to maintain crop productivity in the face of a changing climate. However, despite considerable advances in elucidating the mechanisms of PCD regulation in plants, many gaps in our understanding of this essential process remain (Kacprzyk et al. 2024).

Mitochondria and chloroplast are both implicated in the sensing of cellular stress, initiating the onset of cell death response and PCD execution in plants (Yao and Greenberg 2005; Gao et al. 2008; Kim et al. 2012; Van Aken and Van Breusegem 2015; Mittler et al. 2022; Schwarze et al. 2023). The permeabilization of energy organelles is considered to play a direct role in plant PCD, leading to the generation of high levels of reactive oxygen species (ROS), and the release of death‐promoting mediators, such as cytochrome c and cytochrome f into the cytosol (Van Aken and Van Breusegem 2015). However, mitochondria and chloroplasts can also regulate the cellular responses for death and survival through organelle‐to‐organelle and organelle‐to‐nucleus communication (Van Aken and Van Breusegem 2015; Mittler et al. 2022). In chloroplasts, the oxygenic photosynthesis is not only a driver of carbon assimilation but also a source of signals mediating cell responses and acclimation to environmental, metabolic, or developmental triggers (Mittler 2017; Mhamdi and Van Breusegem 2018; Foyer and Hanke 2022). Photosynthetic capability is an early target of environmental stress conditions, often declining before other cellular functions (Feller 2016; Cardona et al. 2018; Lodeyro et al. 2021), and indeed, chloroplasts are the key sites of excess production of ROS, including singlet oxygen (^1^O_2_), superoxide (O_2_^–^), and hydrogen peroxide (H_2_O_2_) under a range of stress conditions (Mittler et al. 2022). Consequently, modulation of chloroplast redox pathways was a suggested tool for strategies improving plant adaptation to the challenges of global warming, with multiple studies demonstrating that plant stress tolerance can be manipulated using ROS scavengers targeted to chloroplasts (reviewed by Lodeyro et al. (2021)). Better understanding of PCD signaling mediated by ROS and chloroplast oxidative stress responses is therefore required for the development and optimization of these solutions.

Under the high light stress, the flux of photons exceeds the plant energy needs, leading to the production of O_2_^**−**^ and ^1^O_2_ in photosystems I and II of the chloroplast, and H_2_O_2_ in chloroplast stroma and peroxisomes (González‐Pérez et al. 2011; Mittler et al. 2022). Studies of photooxidative stress in plants suggested that ^1^O_2_ is the main ROS involved in the damage of leaf tissues (Triantaphylides et al. 2008), and ^1^O_2_ has been shown to be the major ROS produced from triple excited chlorophyll molecules in the chloroplasts under high light exposure in cultured Arabidopsis cells (González‐Pérez et al. 2011). ^1^O_2_ generated in chloroplasts can act as a toxic molecule that inhibits photosynthesis and cell function, or it can act as a signal that can, depending on the degree of the stress, lead to the initiation of several different cell responses, such as stress acclimation or activation of cell death (op den Camp et al. 2003; Shumbe et al. 2016; Dogra et al. 2018). Much of our understanding of signaling components of cell death pathways triggered by ^1^O_2_ comes from studies of *Arabidopsis thaliana* mutants, such as *flu,* demonstrating a cell death phenotype mediated by EXECUTER proteins (EX1 and EX2) (Kim et al. 2012), and *ch1* (Triantaphylides et al. 2008) where the cell death phenotype is EX1‐independent and instead might be mediated by oxidized derivatives of *β*‐carotene, such as *β*‐cyclocitral and dihydroactinidiolide (Kim and Apel 2013; Ramel et al. 2013; Laloi and Havaux 2015). However, ^1^O_2_ responses in wild‐type plants might show considerable differences and higher complexity compared to mutant backgrounds (Kim and Apel 2013). The ^1^O_2_‐specific photosensitizer Rose Bengal (RB) can be utilized to enhance the production of ^1^O_2_ upon light exposure in wild‐type plants and to study its biological activity in different contexts (Triantaphylides et al. 2008; Kim and Apel 2013; Gutiérrez et al. 2014). However, while numerous studies used RB for studying responses induced by ^1^O_2_ in plants and algae (Fischer et al. 2005; Dominguez‐Solis et al. 2008; Sun et al. 2011; Li et al. 2022; Roach et al. 2022; Percival 2023), using RB to specifically study the PCD pathway induced by ^1^O_2_ received considerably less research attention (Gutiérrez et al. 2014; Koh et al. 2016).

In this study, we used RB to examine features of ^1^O_2_‐mediated PCD in Arabidopsis cell suspension cultures. The cell suspension cultures facilitate PCD induction and modulation using a range of physical and chemical stimuli, and what is relevant in the context of this study, direct comparisons of death response induced in photosynthetic and chloroplast depleted cells (Doyle et al. 2010). Importantly, the cell suspension cultures enable monitoring of cell death progression using light microscopy, which enables differentiation between PCD and uncontrolled necrotic cell death. This distinction is based on the presence of hallmark PCD morphology: protoplast condensation and retraction from the cell wall, which is not observed in the case of cells dying via accidental cell death (necrosis) in response to overwhelming stress levels (Reape et al. 2008; Kacprzyk et al. 2017). Thanks to these features, the cell culture system offers the opportunity to specifically sample plant cells undergoing PCD and to identify the associated changes in gene expression (Burke et al. 2023). Indeed, one of the previous studies on RB‐induced PCD utilized cell suspension cultures to demonstrate the light‐ and chloroplast‐dependent character of the induced cell death program and to generate insights into transcriptional responses to RB in plant cells under light conditions (Gutiérrez et al. 2014). Here, we built upon this work to demonstrate that the PCD induced by RB is dependent on calcium signaling and mitochondrial involvement, thereby showing common features of regulated cell death in plants (Van Aken and Van Breusegem 2015; Bosch and Franklin‐Tong 2024). We also performed RNA‐sequencing on cell cultures treated with RB under different light regimes and treatment conditions, to again demonstrate the light‐ and chloroplast‐dependent character of RB‐induced PCD in cell suspension cultures. The inferred gene regulatory network (GRN) highlighted oxidative stress markers, including those specific to ^1^O_2_ response, and underscored the involvement of pathways commonly implicated in PCD. However, the results also highlight that prolonged exposure to RB may lead to light‐independent toxicity, characterized by features of necrotic cell death, including faster cell death progression, the absence of characteristic PCD morphology and gene expression changes, and underline the role of chloroplasts as key organelles in maintaining cellular homeostasis.

## 2 Material and Methods

### 2.1 Cell Suspension Cultures and Growth Conditions

Previously established *A. thaliana* (ecotype Col‐0) suspension cell culture (Burke et al. 2024) was maintained as described by May and Leaver (1993). Briefly, cells were grown in full‐strength liquid MS medium: 4.3‐g/L basal salts, 0.5‐mg/L 1‐naphthaleneacetic acid (NAA), 0.05‐mg/L kinetin, 3% (w/v) sucrose, pH 5.8; in 250‐mL Erlenmeyer flasks on an orbital shaker set to 120 rpm, at 22°C. All chemicals were purchased from Duchefa, Biochemie. Cells were subcultured weekly by transferring 10‐mL cells into 100 mL of fresh growth medium. Dark‐grown cultures were maintained in constant darkness, and light‐grown cultures were maintained under a constant light intensity of ~45–50 μmol photons m^−2^ s^−1^.

### 2.2 Cell Death Inducing Treatments of Cell Suspension Cultures

Twenty milliliter aliquots of suspension cells in 100‐mL Erlenmeyer flasks were used for experiments. Cell death was induced by treatment with 0.1‐ and 0.5‐μM RB, as described by Gutiérrez et al. (2014); and by heat stress (HS, 51°C, 10 min) performed in a shaking (85 oscillations/min) waterbath (Grant OLS200), as described by Hogg et al. (2011).

### 2.3 Chemical Treatments of Cell Suspension Cultures

Norflurazon (NF), spectinomycin (SM), and lincomycin (LM) treatments (chemicals purchased from Merc Millipore Ireland) were applied at concentrations of 1 μM, 0.2 mM, and 2 mM as described by Doyle et al. (2010) at the time of subculture and subsequently, the cells were grown in light for 7 days prior to cell death induction with 0.1‐μM RB.

All other chemical treatments (3‐MA, CsA, CHX, and LaCl_3_) were applied to 20‐mL aliquots of 7‐day‐old cells in 100‐mL Erlenmeyer flasks as previously described, including respective solvent controls (Doyle et al. 2010; Kacprzyk et al. 2017; Burke et al. 2023). Time of pre‐treatment prior to cell death induction with 0.1‐μM RB, used solvents, and concentrations are indicated in Table S1.

### 2.4 Determination of Rates of PCD, Necrosis, and Viability

The rates of cell viability, PCD and necrosis were determined using a light/fluorescent microscope as described by Hogg et al. (2011). Briefly, only viable cells fluoresce green after staining with 1‐μg/mL fluorescein diacetate (FDA, Sigma‐Aldrich). Dead, FDA‐negative cells were scored as PCD if they displayed protoplast shrinkage or as necrotic if this morphology was absent. A minimum of 200 cells were scored per sample. Statistical analyses were performed using SPSS 22.0 software (IBM SPSS Statistics for Windows).

### 2.5 Determination of Chlorophyl Concentration

Growth medium was separated from the suspension cells using vacuum filtration with Buchner funnel and Whatmann Grade 1 paper. Two grams of fresh cells were ground in 2 mL of acetone using a mortar and a pestle. Subsequently, samples were centrifuged and 400–750 nm absorption spectra of resulting supernatants analyzed using a Varian Cary 300 Bio UV–Visible spectrophotometer. The following formula was used to calculate chlorophyll concentration (μg chlorophyll/g fresh weight) (Mackinney 1941; Arnon 1949): [Chlorophyll] = 20.2(A_645nm_) + 8.02(A_663nm_). Statistical analyses were performed using SPSS 22.0 software (IBM SPSS Statistics for Windows).

### 2.6 RNA Extraction, Quantification, and RNA‐Seq

RNA was harvested after 30 min of treatment with 0.5‐μM RB. Five milliliters of cell suspension were sampled from each flask, growth medium removed via vacuum filtration using a Buchner funnel, and approximately 100 mg of cells transferred to 2‐mL round bottom Eppendorf tubes containing 20 chrome‐steel beads. Samples were flash frozen and stored at −80°C until RNA extraction. The frozen cells were homogenized in a mixer mill (Retsch MM400) for 4 min (20 oscillations/s), and total RNA extracted using the QIAGEN RNeasy Plant Mini kit, including the on‐column DNAse digestion. The resulting total RNA was quantified using Nanodrop (Thermo Scientific) and RNA integrity number estimated using Bioanalyzer (Agilent), with all samples achieving RIN scores of 9 or higher. The library preparation and mRNA sequencing (poly‐A selection, paired‐end 150 bp reads, 6G clean data per sample) were performed by Biomarker Technologies (BMKGene, Münster, Germany). Three independent repeats of the experiment were carried out.

### 2.7 Bioinformatics

The differential gene expression (DGE) analysis, gene ontology (GO) enrichment, and GRN inference were using Galaxy graphical user interface (Afgan et al. 2018) and packages and settings described in Burke et al. (2023). Briefly, FastQC was used for raw reads quality control (Andrews 2010), Trimmomatic for pair reads trimming (Bolger et al. 2014), and HISAT2 (Kim et al. 2019) for mapping to TAIR10 Arabidopsis genome (Lamesch et al. 2012). The expression value counts were obtained using htseq‐count function in HTSeq (Anders et al. 2015), and DESeq2 (Love et al. 2014) was used for DGE analysis with Benjamini and Hochberg false discovery rate correction applied (Benjamini and Hochberg 1995). Functional enrichment analysis was performed using Metascape (Zhou et al. 2019) and String database (Szklarczyk et al. 2021), both using default parameters. For prediction of transcription factors (TFs) with overrepresented targets among identified DEGs, the TF enrichment tool of the Plant Transcriptional Regulatory Map (PlantRegMap) was applied via the motif method and adjusted *p* value cut off 0.05 (Jin et al. 2016). GRN was constructed in Cytoscape (Shannon et al. 2003) using GeneMANIA app (Warde‐Farley et al. 2010), with network clustering carried out using Markov Cluster Algorithm (MCL) with granularity set to 2.5 using in Clustermaker (Morris et al. 2011). For each cluster, the functional enrichment analysis was carried out using Metascape (Zhou et al. 2019) and significantly enriched GO Biological Progresses and KEGG Pathways overlayed over relevant genes on visualization of each cluster. Gene list comparisons were carried out using *phyper* hypergeometric test with false discovery (FDR) correction (Benjamini and Hochberg 1995) applied to *p* values in R (Rstudio Team 2021).

## 3 Results

### 3.1 RB Induces Light‐ and Chloroplast‐Dependent PCD and Demonstrates Dark Toxicity in *A. thaliana* Suspension Cells

**FIGURE 1:**
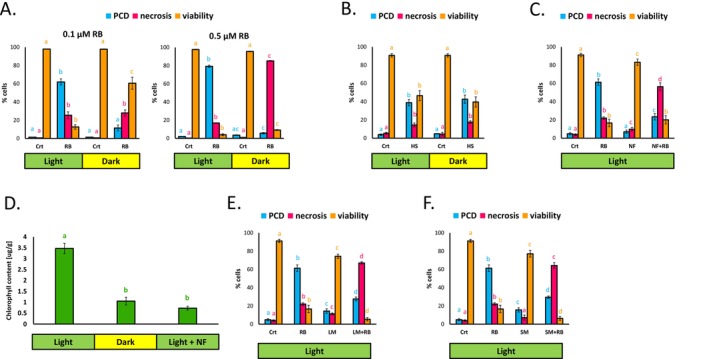
Programmed cell death induced by Rose Bengal in Arabidopsis cell suspension cultures (Col0) is light and chloroplasts dependent. Rates of PCD, necrosis, and viability in light‐ and dark‐grown cells treated with 0.1‐ and 0.5‐μM Rose Bengal (RB) for 24 h (A). Rates of PCD, necrosis, and viability in light‐ and dark‐grown cells 24 h after heat stress (HS, 51°C, 10 min) treatment. This duration and intensity of heat treatment was selected to detect potential shifts toward either pro‐survival or pro‐death response (B). Rates of PCD, necrosis and viability induced by 24‐h treatment with 0.1‐μM RB in cells grown in the light in presence and absence of norflurazon (NF) (C). Chlorophyll content in dark grown cells, and in cells grown in the light in presence and absence of NF (D). Rates of PCD, necrosis and viability induced by 24 h treatment with 0.1 μM RB in cells grown in the light in presence and absence of lincomycin (LM) (E) and spectinomycin (SM) (F). Bars represent the mean of 3 independent repeats of the experiment (± SEM). Different letters indicate statistically significant difference in rates of PCD, necrosis, viability or chlorophyll content between the treatments (ANOVA and Tukey post hoc *p* < 0.05).

The PCD was induced in light‐grown, but not in dark‐grown *A. thaliana* Col‐0 cells after 30 min of treatment with 0.5‐μM RB (Gutiérrez et al. 2014). We further explored the mode of cell death induced by RB and its connection to light and chloroplast function, using a newly established cell line of the same ecotype, and investigating the response to prolonged incubation with RB (up to 24 h). The results recapitulated a previously observed significant increase in rates of PCD (cell death associated with protoplast shrinkage) induced by RB only in light‐grown cells, with higher rates of PCD observed for 0.5 μM compared to 0.1‐μM RB (Figure 1A). Our data also highlighted previously unreported toxicity of RB in plants, that occurred after prolonged exposure, resulting in plant cells undergoing uncontrolled necrosis, with dark‐grown cells appearing more sensitive to this effect at the higher RB concentration tested (0.5 μM), and exhibiting lower rates of viability than light‐grown cells as a result (Figure 1A). In contrast, at lower concentration of RB (0.1 μM) dark‐grown cells exhibited higher viability than light‐grown cells. Light‐dependent differences in cell death response were absent when heat was used as a stressor (Figure 1B). Subsequent experiments convincingly suggested that the difference in response of light and dark‐grown cells to RB stress treatment is linked to the presence of functional chloroplasts. The 0.1‐μM RB (resulting in viability drop to ~20% in light‐grown cells) was used to facilitate detecting potential shifts toward either pro‐survival or pro‐death response. Cells grown in the light in the presence of norflurazon (NF) showed reduced levels of PCD induced by 0.1‐μM RB, with cells undergoing necrosis instead (Figure 1C). NF is an herbicide that can inhibit carotenoid production and suppress chloroplast development and function (Mayfield et al. 1986; Strand et al. 2003). Consequently, NF‐treated light‐grown cells contain chlorophyll levels comparable to that of dark‐grown cells (Figure 1D). Further, use of antibiotics spectinomycin (SP) and lincomycin (LM), which inhibit chloroplast protein synthesis (Thomson and Ellis 1972; Mulo et al. 2003), resulting in beige‐colored cultures deprived of functional chloroplasts (Doyle et al. 2010) had a similar effect on the RB‐induced cell death response in light‐grown cells (Figure 1E,F). Interestingly, light‐grown cells, where chloroplast function or development was inhibited by chemical treatment (NF, SP, and LM), demonstrated responses to 0.1‐μM RB resembling that of dark‐grown cells treated with higher RB concentration (0.5 μM), with lower levels of PCD, but higher rates of necrosis, and no increases in cell survival. This can point out not only to an essential role of chloroplasts in RB‐induced signaling involved in PCD but also to potential differences in cytoprotective signaling pathways between photosynthetic and non‐photosynthetic cells.

### 3.2 RB‐Induced Phototoxicity Is Associated With Typical Features of Plant PCD Occurring in Arabidopsis Suspension Cells

**FIGURE 2:**
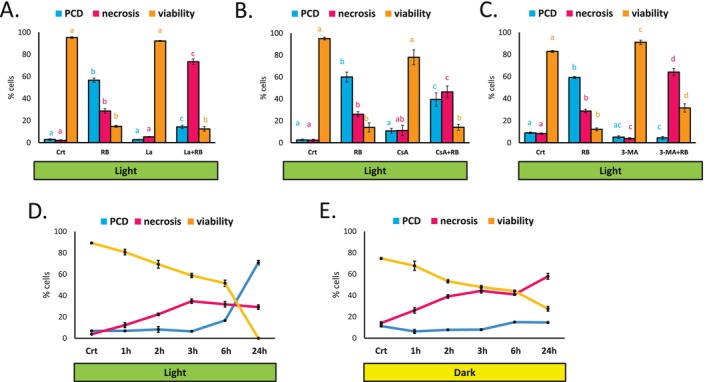
Features of PCD induced by Rose Bengal (RB) in *Arabidopsis thaliana* suspension cells. Rates of PCD, necrosis and viability in light‐grown cells after 24 h treatment with 0.1‐μM RB: in presence and absence of calcium channel blocker LaCl_3_ (La) (A); with and without 24‐h preincubation with cyclosporin A (CsA) (B), with and without 24‐h preincubation with 3‐methyladenine (3‐MA) (C). Bars represent the mean of 3 independent repeats of the experiment (± SEM). Different letters indicate statistically significant difference in rates of PCD, necrosis and viability between the treatments (ANOVA and Tukey post hoc *p* < 0.05). Time course of PCD, necrosis, and viability changes induced by treatment with 0.5‐μM RB in light (D) and dark (E) grown cells. Error bars are ± SEM of 3 independent experiments.

To further characterize the cell death program induced by RB in light‐grown cells, we used chemical treatments previously reported to modulate PCD by targeting different elements of signaling pathways involved. Lanthanum chloride (LaCl_3_) is a widely used extracellular calcium channel blocker (Lewis and Spalding 1998) and has been shown to inhibit PCD in plant cell suspension cultures induced by heat, ethanol, heat, staurosporine, or hydrogen peroxide (McCabe et al. 1997). In this study, co‐treatment with RB and LaCl_3_ resulted in a significant decrease of PCD rates induced after 24 h compared to treatment with RB alone, with cells undergoing necrosis instead (Figure 1A), indicating that PCD associated with RB phototoxicity depends on calcium signaling. Further, we probed the mitochondrial involvement, a well‐established element of the PCD transduction pathway in plants (Van Aken and Van Breusegem 2015), using an inhibitor of mitochondrial permeability transition, cyclosporin A (CsA), previously demonstrated to inhibit PCD in plant cells (Contran et al. 2007; Kacprzyk et al. 2017). Cells pre‐incubated with CsA before treatment with RB exhibited lower rates of PCD, with necrosis occurring instead (Figure 2B). Another chemical treatment that effectively blocked RB‐induced PCD, resulting mostly in induction of secondary necrosis and slightly increased viability, was 3‐methyladenine (3‐MA) (Figure 2C). The 3‐MA is a widely used autophagy inhibitor (Takatsuka et al. 2004; Kosic et al. 2021), but it was also reported to inhibit mitochondrial permeability transition (Xue et al. 2002) and consequently PCD induced in plants in a manner independent of autophagy inhibition (Kacprzyk et al. 2014). Overall, the data indicate that RB‐induced PCD likely depends on calcium signaling, with at least some level of mitochondrial involvement. Finally, due to previously reported differences in timing between PCD and necrosis (Kacprzyk et al. 2017), we examined the time course of changes in PCD, necrosis and viability levels induced by RB in both light‐ and dark‐ grown cells (Figure 2D,E). The rates of protoplast shrinkage associated PCD, induced in light grown cells, were increased after 6 h of treatment, with the majority of cells having died via this mode after 24 h. In contrast, rates of necrosis induced by RB treatment in both dark and light grown cells started to increase at earlier timepoints.

### 3.3 PCD Induced by RB Is Associated With Distinct, Early Transcriptional Reprogramming, Unlike Necrosis Caused by Light‐Independent Toxicity of RB

**FIGURE 3:**
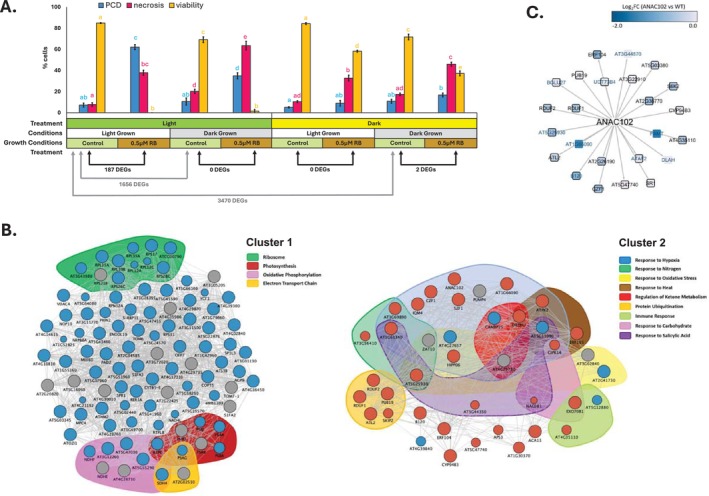
Both light and chloroplasts are required for induction of PCD by Rose Bengal, that is associated with transcriptional reprogramming in *Arabidopsis thaliana* suspension cultures. (A) Dark‐ grown and light‐grown cells were treated with 0.5 μM RB either in the light or in the dark, resulting in four different growth conditions—treatment conditions combinations ([1] light grown cells treated in the light; [2] dark grown cells treated in the light; [3] light grown cells treated in the dark; [4] dark grown cells treated in the dark). Three independent repeats of the experiment were carried out. Rates of PCD, necrosis and viability for each treatment were recorded after 24 h. Bars represent the mean (± SEM). Different letters indicate statistically significant difference in rates of PCD, necrosis and viability between the treatments (ANOVA and Tukey post hoc *p* < 0.05). The RNA has been harvested after 30 min of treatment for sequencing and DGE analysis. The number of genes transcriptionally responsive to RB under each grown condition—treatment condition combination is indicated (DEGs = differentially expressed genes), as well as number of DEGs between dark‐ and light‐grown cells, treated in the light and in the dark. (B) GRN underlying the RB‐induced PCD. The clustered GRN was constructed using GeneMANIA using 187 genes responsive to RB (listed in Table SS2). Red nodes represent genes upregulated and blue nodes downregulated by 30 min treatment with RB. Result nodes (inserted by GeneMANIA) are colored gray, and the node size represents the connectivity of each node (the more highly connected nodes appearing larger). Edge thickness represents predicted strength of the interaction. Gene enrichment was carried out separately for each cluster using Metascape, and genes were highlighted based on the significant gene enrichment terms to which they contribute. Detailed information on the GRN nodes, their connectivity and enriched terms is provided in Table SS4. (C) RB‐induced genes regulated through ANAC102. Blue nodes indicate genes downregulated in *ANAC102* OE under MV stress and/or upregulated in *anac102* KO under control conditions (Luo et al. 2024) with the color representing the log_2_FC (or negative log_2_FC for *anac102* KO). Black node border indicates that the target gene was identified in ChIP‐Seq analysis of ANAC102 (Song et al. 2016). Blue node label indicates ANAC102—target gene interaction predicted by a previous GRN study (De Clercq et al. 2021).

To further characterize the response to RB and its relationship with the presence of functional chloroplasts and the used light regime, we treated light‐ and dark‐ grown cells with 0.5 μM RB either in light or in the dark (Figure 3). The rates of PCD, necrosis and viability were determined after 24 h. The highest rates of PCD were observed in light‐grown cells, treated in the light; with dark‐grown cells treated in the light showing smaller, but statistically significant, increase in PCD levels, likely due to development of some functional chloroplasts during the 24‐h treatment in the light. No increases in PCD levels were observed when either light‐ or dark‐grown cells were treated in the dark. A significant light‐independent cytotoxicity manifesting as necrosis was observed for all treatments with RB, with the highest rates induced by RB in dark‐grown cells treated in the light. Interestingly, when treatment was performed in the dark, the light‐grown cells exhibited lower rates of necrosis, and significantly higher viability than the dark‐grown cells. To identify gene expression changes associated with early response to RB, cells from all treatments were sampled for transcriptome profiling (paired‐end mRNA sequencing) after 30 min of treatment and DGE analysis carried out (Table S2). Surprisingly, at this time point, application of RB induced significant changes in gene expression only in light‐grown cells treated in the light, corresponding to treatment with the highest levels of PCD observed after 24 h, with 187 differentially expressed genes identified (40 upregulated DEGs, 147 downregulated DEGs). There was an overlap of 24 genes between 187 DEGs identified here, and the 1705 DEGs from the microarray experiment from (Gutiérrez et al. 2014). The subsequent gene enrichment analysis of this gene list highlighted terms related to cellular response to hypoxia, salt stress, photosynthesis and oxidative phosphorylation, and cellular components including chloroplast/plastid, ribosome and mitochondrion (Table S3A). As expected, prominent differences were observed between transcriptomes of light‐ and dark‐grown cells, with 3470 genes differentially expressed between control cells for treatments performed without change of light regime, that is, control light‐grown cells for light treatment versus control dark‐grown cells for dark treatment (Figure 3 and Table S2C). This number of DEGs was reduced by ~50% (to 1656) after dark‐grown cells were incubated in the light for 30 min (Table S2D), indicating rapid light‐induced transcriptional reprogramming induced by light. The GO enrichment analyses comparing the gene expression profile in light‐ and dark‐grown cells underscored terms related to photosynthesis, plastid organization and carbon fixation, but also multiple clusters of terms associated with response to diverse range of stimuli (Table S3b,c), suggesting differences in basal level of stress signaling pathways. Further, the GRN clustered using MCL algorithm was constructed to facilitate identification of highly connected hub signaling genes, likely to play a central role in the PCD pathway induced by RB. Gene enrichment analysis was carried out on two identified GRN clusters, highlighting biological processes relevant to PCD programs induced (network topology, including genes from both clusters and their connectivity, and enriched terms are listed in Table S4A–D). Additionally, we compared the list of DEGs responsive to RB in this study, with genes regulated in Arabidopsis suspension cells under PCD‐inducing conditions (Burke et al. 2023). The outcomes of these comparisons, with FDR‐corrected hypergeometic p‐values, are provided in Table S5A. The results suggested significant overlap between genes differentially expressed in response to PCD‐inducing RB treatment and transcriptional response to PCD induction by heat (abiotic stress) and salicylic acid (mimicking biotic stress), but not by critical dilution that triggers cell death through withdrawal of social signaling between cells, rather than applied stress, and thus is similar to developmental PCD program (Burke et al. 2023). The overlap between response to RB from this study and response to methyl viologen (MV) (Luo et al. 2024), another chemical generating superoxide and causing chloroplast damage under light conditions (Gadjev et al. 2006), was also significant (25/187 RB responsive genes, hypergeometric *p* value = 0.00013). Second, as the constructed GRN (Cluster 2) featured *ANAC102*, encoding a TF mediating chloroplast retrograde signaling (D'Alessandro et al. 2018), we compared the RB‐induced genes to ANAC102 regulome based on RNA‐seq data of ANAC102 overexpressor and knockout lines (Luo et al. 2024), ChIP‐Seq (Song et al. 2016) and GRN prediction data (De Clercq et al. 2021), revealing 24 genes upregulated by RB that might be under negative regulation of ANAC102 (Figure 3C and Table S6).

## 4 Discussion

Presented results advance our understanding of RB‐induced cell death in plants and support the use of this photosensitizer for targeted induction of chloroplast‐mediated PCD in photosynthetic cells. In our system, PCD induced by RB was dependent on light, and on the presence of functional chloroplasts, in line with previous research investigating the effect of short‐term (30 min) treatment with RB on *A. thaliana* cell suspension cultures (Gutiérrez et al. 2014). Consistently, the light dependent tissue death is mediated by chloroplast ^1^O_2_ in mutant backgrounds including *crumpled leaf* (*crl*) (Li et al. 2020) and *chlorina1* (*ch1*) (Ramel et al. 2013) (high light stress) and *flu* (dark‐to‐light transition) (Meskauskiene et al. 2001; Kim et al. 2012). Here, prolonged incubation with RB induced PCD only when treatment was performed in the light, with cells treated in the dark exhibiting higher viability; in contrast to heat stress response, where PCD‐viability threshold did not differ between light‐ and dark‐grown cells. However, prolonged treatment with RB‐resulted also in significant increase in rates of uncontrolled death (necrosis), under both light and dark conditions. The kinetics of both cell death types induced by RB differed, with necrosis occurring more rapidly, and development of PCD taking between 6 and 24 h, a timespan comparable to that of PCD induced by other chemicals and abiotic stimuli (Reape et al. 2008).

The light‐independent toxicity of RB has not been previously described in plants. However, there are reports suggesting that in addition to its phototoxicity, this drug has also an intrinsic cytotoxicity in absence of light in different cancer cell lines (Mousavi et al. 2009; Karthikeyan et al. 2011; Nascimento et al. 2013), inducing predominantly necrotic cell death that appeared independent of ROS generation (Mousavi et al. 2006). Photoactivated RB was also reported to induce both apoptosis and necrosis in primary corneal endothelial cells (Cho et al. 1999). These findings highlight that the mechanisms underlying cellular response to RB are often complex, depending on its concentration, timing of treatment, presence of illumination and cell type. However, this complexity has not been broadly investigated in plants and needs to be considered while designing studies using this photosensitizer. Further work is required to identify the mechanisms underlying RB's dark toxicity in plants. This could include investigation of its direct interactions with cellular components, as previous studies shown that dark toxicity of RB can be reduced by encapsulation of the drug (Karthikeyan et al. 2011).

Interestingly, when the development of functional chloroplasts was inhibited in light‐grown cells through the use of NF, SP, and LM (Thomson and Ellis 1972; Mayfield et al. 1986; Mulo et al. 2003; Strand et al. 2003), the RB‐induced PCD was blocked, but the loss of viability was not prevented, with secondary necrosis occurring instead. The chloroplast, therefore, appeared to be the organelle essential for generation and response to the PCD‐inducing signal produced via RB. Chlorophyll is the source of the ^1^O_2_ driving the chloroplast‐induced cell death in the light (Koh et al. 2016). However, RB has been previously shown to localize not only to chloroplasts (Gutiérrez et al. 2014) but also to the plasma membrane (Gutiérrez et al. 2014; Koh et al. 2016) and indeed, the formation of ^1^O_2_ induced by RB is not dependent on chloroplasts only, as it was previously observed in non‐photosynthetic root tissue (Mor et al. 2014). The ^1^O_2_ signaling mediates plant acclimation and PCD responses to environmental changes that affect photosynthetic function (Kim et al. 2012) with photosynthetic ROS production being a major driving force in chloroplast‐to‐nucleus retrograde signaling (Foyer and Hanke 2022). In addition, the direct oxidative action of ^1^O_2_ may lead to the loss of viability due to the disruption of plasma membrane integrity (Koh et al. 2016). It is possible that the balance between two responses has been shifted toward this direct toxicity in the light‐grown cells deprived of functional chloroplasts via chemical treatment. Further, the chloroplast antioxidant systems can either propagate ROS signals, including the singlet oxygen, or detoxify excess ROS (Foyer and Hanke 2022). It is therefore possible that in the absence of functional chloroplasts, ^1^O_2_ produced by RB photoactivation in other cellular compartments would fail to activate the genetically controlled PCD signaling and instead cause viability loss due to direct, uncontrolled oxidative damage, as observed here following treatment with NF, SP, and LM. Indeed, the transcriptional profiling we carried out highlighted differences between light‐ and dark‐grown cells in basal expression of genes belonging to GO terms for responses to an array of different stress types, including oxidative stress and detoxification pathways. The marked differences in antioxidative machinery, both in terms of activity and subcellular location, were also previously observed between photosynthetic and non‐photosynthetic leaf tissues of variegated Pelargonium plants (Vidović et al. 2016). The fact that the rates of necrosis induced by 0.5 μM RB were lower in light‐ compared to dark‐grown cells, regardless of whether the treatment itself was performed in the presence or absence of light, could indirectly indicate a link to cells' antioxidant capacity and ability to dampen the direct oxidative damage to the plasma membrane or other components, rather than differences in signaling pathways related to photosynthetic activity. In contrast, the cell death induced by RB in chloroplast‐containing cells under light conditions showed typical features of genetically regulated PCD previously characterized in Arabidopsis suspension cells and other models for studying plant PCD: dependence on calcium signaling and mitochondrial involvement (Kacprzyk et al. 2011). Application of the extracellular calcium channel blocker LaCl_3_ (Evans and Evans 1990), which previously blocked hallmark features of stress‐induced PCD, including early DNA fragmentation and protoplast shrinkage in Arabidopsis cells (McCabe et al. 1997; Kacprzyk et al. 2017; Schwarze et al. 2023), effectively inhibited PCD induced by RB, suggesting that it is an active, calcium‐driven process. Likewise, application of CsA, a blocker of mitochondrial permeability transition, resulted in decreased PCD levels induced by RB, as previously shown for PCD induced by both abiotic and biotic stimuli (Tiwari et al. 2002; Contran et al. 2007; Gao et al. 2008; Kacprzyk et al. 2017). It needs to be highlighted that CsA can also inhibit chloroplast cyclophilins (Breiman et al. 1992), and in this way, potentially exert its effect via direct modulation of chloroplast function. However, the mitochondrial involvement was further underscored by the reduction of the RB‐induced PCD rates after pre‐treatment with 3‐MA. While broadly used as an autophagy inhibitor (Takatsuka et al. 2004; Kosic et al. 2021), 3‐MA was also reported to block mitochondrial permeability transition (Xue et al. 2002) and inhibit both mitochondrial swelling and PCD induced by salicylic acid, in a manner independent of its effects on autophagy (Kacprzyk et al. 2014) and possibly related to its recently reported effects on the expression of mitochondrial genes and mitochondrial stress signaling regulome (Burke et al. 2023). The effect of 3‐MA on PCD exerted via autophagy inhibition in the current study cannot be excluded, but taken together, these results suggest that the chloroplast‐dependent PCD induced by RB is calcium‐driven and mediated by mitochondrial events.

The ^1^O_2_, generated either during photosynthetic stress or in response to treatment with photosensitizers such as RB (Gutiérrez et al. 2014) can cause extensive photodamage by readily reacting with macromolecules in its vicinity but also contributes to chloroplast‐to‐nucleus retrograde signaling, priming acclimation and cell death responses (Dogra et al. 2018). When we investigated the early (30 min) transcriptional response to RB, only conditions resulting in high rates of PCD (light treatment of light‐grown cells) were characterized by significant changes in gene expression, whereas treatments associated with predominantly light‐independent toxicity (necrosis) of RB did not induce rapid transcriptional responses. Indeed, the RB‐induced changes in gene expression showed a significant overlap with the transcriptional response to heat (abiotic stress) or salicylic acid (mimic of biotic stress) previously characterized in Arabidopsis cell suspension cultures (Burke et al. 2023), highlighting commonalities in the transcriptional response to PCD‐inducing levels of stress. The response to RB observed here in suspension cells also significantly overlapped with the response of Arabidopsis seedlings to MV, another chemical agent causing oxidative stress in the chloroplasts (Gadjev et al. 2006). Similarly to previous examinations of RB‐induced transcriptional changes using microarrays (Gutiérrez et al. 2014), we found no evidence for the involvement of EXECUTER‐dependent signaling, known to mediate the cell death phenotype of the *flu* mutant (Kim et al. 2012). Construction of the GRN yielded further insights into the cellular response to PCD‐inducing levels of RB. The GRN featured two main clusters. Cluster 1 was composed mostly of genes downregulated in response to RB, enriching terms related to photosynthesis, energy production, and ribosomal function. In line with this, one of the RB‐downregulated genes from Cluster 1 was *PRIN2* (*PLASTID REDOX INSENSITIVE* 2), which encodes a chloroplast component involved in redox‐mediated retrograde signaling that synchronizes the expression of photosynthetic genes from nuclear and plastid genomes under excess light (Kindgren et al. 2012). These observations may indicate that the mechanisms sustaining primary metabolism are switched off in response to PCD‐inducing levels of photodamage caused by ^1^O_2_. In contrast, Cluster 2 of the RB‐responsive GRN contained predominantly upregulated genes, enriching GO terms related to stress response signaling, including oxidative stress, hypoxia, immune response, and other conditions known to induce cell death in plants. Similar enriched terms were highlighted for the GRN constructed for the core PCD pathway in Arabidopsis cell culture (Burke et al. 2023) and by spatiotemporal transcriptome analyses of immune cell death in Arabidopsis (Salguero‐Linares et al. 2022). The most connected, central hub gene in Cluster 2 was *SZF1*, previously used as a ^1^O_2_ response marker (Gutiérrez et al. 2014), although *SZF1* transcription can also be induced by MV and ozone. Further, six other genes (*ERF105, ATL2, RDUF1, EXO70B1, AT4G27657,* and *AT1G30370*) from Cluster 2 were previously identified as exclusively responsive to ^1^O_2_ (Gadjev et al. 2006), overall suggesting that the induced transcriptional response, and putatively also the resulting PCD, were indeed mediated by ^1^O_2._ Two ethylene‐responsive TFs from the GRN, *ERF104* and *105*, have been shown previously to be involved in the fast retrograde signaling response and acclimation response to high light (Moore et al. 2014; Vogel et al. 2014) and play a role in plant immunity (Bethke et al. 2009; Cao et al. 2019). *ZAT10* is another example of a TF from Cluster 2 that was previously implicated in the regulation of the expression of reactive oxygen‐induced transcripts (Mittler et al. 2006). Cluster 2 also involved *ANAC102*, encoding a TF identified as a key player in β‐cc‐mediated chloroplast retrograde signaling (D'Alessandro et al. 2018). This TF was first identified as an inducer of detoxification response and plant acclimation to photooxidative stress (D'Alessandro et al. 2018), with recent research suggesting that it may act as both an activator and repressor of stress responses, depending on the stress type and level and promoter context (Luo et al. 2024). In addition to being implicated in the regulation of plant responses to oxidative stress (Luo et al. 2024) and hypoxia (Christianson et al. 2009), ANAC102 has been recently suggested to trigger ROS accumulation and cell death during petal abscission (Furuta et al. 2024). A downstream target of ANAC102, *NAC081* (D'Alessandro et al. 2018), was also included in Cluster 2 of the constructed GRN. The link between ANAC102 and the modulation of ^1^O_2_ induced PCD is further supported by the upregulation of this TF in the *crl* mutant that shows localized cell death (Li et al. 2020), and the fact that *anac102* mutants were sensitive to excessive light due to incomplete chloroplast retrograde signaling and showed increased leaf bleaching (D'Alessandro et al. 2018).

Analysis of previously generated data of ANAC102‐regulated genes (Song et al. 2016; De Clercq et al. 2021; Luo et al. 2024) further underscored a potential link between the ANAC102 regulome and transcriptional responses to RB. This link needs to be further elucidated by future studies involving multi‐timepoint transcriptome profiling to determine RB‐induced changes in gene expression in both wild type and *ANAC102* mutant or gain‐ and loss‐of‐function transgenic lines for time‐resolved analysis of cellular response underlying RB‐induced PCD. Finally, a careful examination of cell death rates and the time course of the cell death process induced by RB in different cell types (both photosynthetic and chloroplast‐free) of *ANAC102* overexpressor and knockout lines is needed in order to functionally validate the role of this TF in cell death and survival decisions in response to singlet oxygen stress in plants.

## 5 Conclusions

The Arabidopsis cell suspension culture system provided a controlled model for dissecting chloroplast‐dependent PCD triggered by RB. It enabled direct, quantitative comparisons between chloroplast‐containing and chloroplast‐depleted cells. The homogeneity of the cultured cells, ease of pharmacological treatments, and microscopic imaging allowed precise, time‐resolved analysis of cell death dynamics and distinction between PCD and necrosis. The obtained results support the view that chloroplasts, under light conditions, play a protective role via maintenance of metabolism and high capacity of antioxidant systems, but can also participate in induction of PCD through ^1^O_2_—mediated signaling pathways (Hoeberichts et al. 2013; Van Aken and Van Breusegem 2015). Based on the obtained data, RB appears as a useful pharmacological tool for investigating ^1^O_2_‐induced, chloroplast‐dependent PCD, which is associated with common features of regulated cell death in plants: early transcriptional reprogramming, dependency on calcium signaling and mitochondrial involvement, and development of protoplast shrinkage morphology within 6–24 h. However, the light‐independent toxicity of RB, in this study manifesting features of necrotic cell death (occurring faster than PCD, without characteristic PCD morphology and changes in gene expression), will need to be considered in future experimental work. Nonetheless, validation of these findings in whole plants will be essential to understand their relevance in the context of plant physiological responses to stress.

## Author Contributions

Study idea was conceived by P.F.M. and further developed by all authors. Experimental design: P.F.M., J.K., Y.J., and R.B. Wet lab experimental work: J.N. Bioinformatics: R.B. and I.C. Data analysis: Y.J., R.B., I.C., and J.K. The final version of the manuscript was developed and approved by all authors (Y.I., R.B., I.C., J.K., and P.F.M.).

## Conflicts of Interest

The authors declare no conflicts of interest.

## Peer Review

The peer review history for this article is available in the Supporting Information for this article.

## Supporting information


**Data S1:** Peer Review.

**Table S1:** Details of chemical treatments used to modulate rates of PCD induced by RB.
**Table S2:** Results of differential gene expression analysis.
**Table S3:** Results of gene enrichment analyses.
**Table S4:** Details of constructed GRN underlying response to RB.
**Table S5:** Overlap between DEGs responsive to RB and genes regulated by other PCD and oxidative stress inducing treatments.
**Table S6:** Overlap between RB‐induced genes and ANAC102 regulome.

## Data Availability

RNA‐seq datasets and associated metadata were uploaded to the NCBI Sequence Read Archive under the Bioproject Accession number PRJNA1111441.
