# Supplementary material for: Rose Bengal Is a Precise Pharmacological Tool Triggering Chloroplast‐Driven Programmed Cell Death in Plants, Dependent on Calcium and Mitochondria, and Associated With Early Transcriptional Reprogramming
Source: Plant Direct. 2025 Oct 20;9(10):e70110. doi: 10.1002/pld3.70110 (PMC12536220; doi:10.1002/pld3.70110)
Supplement: Supplementary file 1 — Data S1: Peer Review. [file PLD3-9-e70110-s002.pdf]

Plant Direct
Open Access

Rose Bengal is a precise pharmacological tool triggering chloroplast driven programmed cell death in plants, dependent on calcium and mitochondria, and associated with early transcriptional reprogramming

Dr. Joanna Kacprzyk, Yasmine Jnaid , Rory Burke , Prof. Inge De Clercq , Dr. Paul F. McCabe

Decision Letter Round 1:

June 19, 2025

Dr. Joanna Kacprzyk

University College Dublin

Dublin 4

Ireland

RE: Rose Bengal is a precise pharmacological tool triggering chloroplast driven programmed cell death in plants, dependent on calcium and mitochondria, and associated with early transcriptional reprogramming

Dear Dr. Kacprzyk:

Thank you for submitting to Plant Direct. All required reviews have been returned and we have now finished our evaluation of your manuscript. In light of the reviewers' and editor's comments, further revisions are needed before the paper can be accepted for publication in Plant Direct.

Please view the editors' and reviewers' comments below and use their suggestions as a guide while you work on your revision.

When uploading the revised version of this article, please be sure to include the following:

-A word document that contains your response to the reviewers. You should respond to each reviewer comment and note the changes made to the manuscript. If you do not agree with a reviewer's comment and choose not to make a suggested revision, please explain why. Please try to provide as complete an answer as possible to each reviewer's criticisms in the "response to reviewers" section.

-A tracked changes document

- A clean version of the latest version of the manuscript

The MS has been reviewed by two reviewers with comments for improvement. Lack of Functional Validation for ANAC102 is a point raised by both reviewers. Kindly address all the comments of the reviewers, and we look forward to the revised version of the MS

To upload your revision, please click the link below.

https://plantdirect.msubmit.net/cgi-bin/main.plex?el=A5Lr1nl5A3pMu2I2A9ftdZYmlwiEgquaeDUVBVJCfwZ

In order to provide as timely a service as possible, we ask that your revision is resubmitted within three months after receipt of this request. If an extension is needed, please send a request, along with a brief explanation, to the editorial office at plantdirect@wiley.com .

Thank you very much for giving us an opportunity to review your work. I look forward to receiving the next version.

Sincerely,

Jyothilakshmi Vadassery

-- Editor comments:

The MS has been reviewed by two reviewers with comments for improvement. Lack of Functional Validation for ANAC102 is a point raised by both reviewers. Kindly address all the comments of the reviewers, and we look forward to the revised version of the MS

-- Reviewer comments:

Reviewer #1:

Jnaid and coworkers investigated the impact of Rose Bengal (RB), a photosensitizer as a precise pharmacological tool that can induce singlet oxygen activated and chloroplast-driven PCD in Arabidopsis thaliana cell suspension cultures in a light dependent manner. This PCD pathway is involves transcriptional reprogramming and requires calcium burst and mitochondrial assistance. Authors also showed that chemical inhibitors such as norflurazon, spectinomycin, or lincomycin inhibits this PCD but leads to necrosis. Calcium signalling inhibitor lanthanum chloride, and mitochondrial membrane protector cyclosporin A, also inhibits this PCD. Transcriptional changes within 30 minutes of RB treatment, revealed ANAC102 as a hub regulating a gene network driving this response.

Overall, the study leads to an understanding of oxidative stress response in cell suspension cultures, which may find applications in enhancing plant resilience to environmental stressors.

However, the impact on live plants could have been much better to deduce such conclusions.

Specific comments:

1. The study could have been extrapolated in live plants as RB treatment is always a challenge in live plants compared to individual cells.

2. Most if these finddings are well established in terms of singlet oxygen triggered and chloroplast driven PCD, Please refer to work done on flu, chlorina 1 and crumpled leaf mutants under dark-to-light transitions and high light stresses

(relevant publications https://doi.org/10.1105/tpc.112.100479, https://doi.org/10.1105/tpc.18.00578, https://doi.org/10.1105/tpc.20.00016)

3. ANAC102 is known to regulate a network of genes involved in xenobiotic responses mainly detoxification and not PCD (work on chlorina 1and crumpled leaf mutants; https://doi.org/10.1105/tpc.18.00578, https://doi.org/10.1105/tpc.20.00016). It's precise role in

PCD is not established, it could have been tested using mutant and overexpression lines under RB treatments.

Reviewer #2:

The manuscript presents a compelling and well-structured study investigating the use of Rose Bengal (RB) as a photosensitizer to induce chloroplast-driven programmed cell death (PCD) in Arabidopsis thaliana cell suspension cultures. The findings have important implications for understanding ROS-induced PCD and plant stress responses. However, there are several areas that require clarification and improvement before the manuscript can be considered for publication.

Introduction:

• The rationale for using Arabidopsis cell suspension cultures is not clearly explained. Including a brief justification (e.g., suitability for microscopy, uniform responses, ease of chemical treatment) would help non-expert readers understand the advantages of this model system.

• The Introduction focuses heavily on singlet oxygen ($^1O_2$) and chloroplast-derived ROS, while other well-established PCD triggers (e.g., developmental cues, pathogen response, abiotic stress) are underrepresented. A more balanced overview would improve the context of the study.

• A brief overview of the general concept of PCD in plants would enhance clarity, particularly for a broader readership.

• Potential limitations of RB, such as its light-independent toxicity, are not acknowledged in the Introduction. This should be briefly mentioned to present a balanced view from the outset.

• The roles of mitochondria and calcium signaling in PCD are central to the study's findings but are not adequately introduced. Including a few sentences about their relevance would better connect the Introduction to the Results.

Results:

• The results convincingly show that RB induces light- and chloroplast-dependent PCD, with higher PCD rates observed at 0.5 µM compared to 0.1 µM. This is well supported by the use of chloroplast inhibitors (norflurazon, spectinomycin, lincomycin) and chlorophyll measurements (Fig. 1A-F).

• A novel and interesting finding is the light-independent (dark) toxicity of RB, particularly in dark-grown cells, which leads to necrosis. This adds originality to the study.

• Calcium and mitochondrial signaling are thoroughly investigated using pharmacological inhibitors ($LaCl_3$, CsA, and 3-MA), and the time-course experiments are well designed, aligning with known PCD pathways.

• The transcriptomic analysis reveals 187 differentially expressed genes (DEGs) associated with stress response and metabolic shutdown. The gene regulatory network (GRN) highlights ANAC102 as a potential key regulator.

Weaknesses and Suggestions for Improvement:

1. Dark Toxicity Mechanism:

The mechanism behind RB's light-independent dark toxicity is not explored in depth. Further investigation or at least a discussion of possible pathways would improve the understanding of RB's non-specific effects.

2. Limited RB Concentration Range:

Only two concentrations of RB (0.1 µM and 0.5 µM) were tested. Including additional concentrations would strengthen the analysis of dose-dependent effects. The authors should consider expanding this range or providing justification for the chosen doses.

3. Single-Time-Point Transcriptomics:

RNA-seq was conducted at a single time point (30 minutes post-treatment), which likely captures only early transcriptional changes. The manuscript should clarify the reasoning behind this time point selection. Additionally, it is not clear how many independent biological replicates were included in the RNA-seq experiment-this should be explicitly stated to evaluate data robustness. To gain further insight into temporal gene regulation, it is recommended to perform RT-qPCR validation of ANAC102 and other key GRN genes at additional time points (e.g., 1 h, 6 h, 12 h, and 24 h).

4. Lack of Functional Validation for ANAC102:

While ANAC102 is highlighted as a key node in the GRN, the manuscript lacks any experimental validation. Even preliminary functional assays would significantly strengthen the claim of ANAC102's regulatory role in RB-induced PCD.

Decision Letter Round 2:

August 11, 2025

Dr. Joanna Kacprzyk

University College Dublin

Dublin 4

Ireland

MSID: 2025-01758-TWR1

MS TITLE: Rose Bengal is a precise pharmacological tool triggering chloroplast driven programmed cell death in plants, dependent on calcium and mitochondria, and associated with early transcriptional reprogramming

Dear Dr. Joanna Kacprzyk:

I am pleased to inform you that your manuscript "Rose Bengal is a precise pharmacological tool triggering chloroplast driven programmed cell death in plants, dependent on calcium and mitochondria, and associated with early transcriptional reprogramming" has been accepted for publication in Plant Direct.

Plant Direct
Open Access

Your article will appear online in the next available issue of Plant Direct. To ensure your article gets published as quickly as possible, please pay attention to the steps detailed below. We have found that most of the delays happen at this stage, especially at the payment stage, so please respond as quickly as possible when prompted.

License Agreement: Once your article has been accepted it will move to Production and undergo admin and file checking - you may receive an email with any queries we have at this stage. When all required items are received by the publisher and queries resolved, the corresponding author will receive an email from Wiley's Author Services system which will ask them to log on at https://authorservices.wiley.com/bauthor and will present them with the appropriate license for completion. Your article cannot be published until both the signed license agreement and payment of the article fee have been received.

Payment of the Open Access Article Publication Fee: All articles published in Plant Direct are fully open access: immediately and freely available to read, download and share. Plant Direct charges a publication fee to cover the publication costs. The corresponding author for this manuscript should have already received a quote from the payments team (cs-openaccess@wiley.com) with the estimated article publication fee; please email cs-openaccess@wiley.com if this has not been received. The corresponding author should log on to the Wiley Author Services site, where the publication fee can be paid by credit card or an invoice. Pro Forma can also be requested. Payment of the publication charge must be received before the article will be published online.

Proofs: You will have the opportunity to look over your paper once more when you receive the author proofs for your article. The proofs will be with you in approximately two weeks. Please note that, in addition to publishing reviewer comments, the author's responses to review comments will also be published alongside the final version of the paper. If you would not like the author's responses to be published, please contact the editorial office at plantdirect@wiley.com .

Promotion of your article: You can help your research get the attention it deserves! Check out Wiley's free Promotion Guide for best-practice recommendations for promoting your work at www.wileyauthors.com/eeo/guide. And learn more about Wiley Editing Services which offers professional video, design, and writing services to create shareable video abstracts, infographics, conference posters, lay summaries, and research news stories for your research at www.wileyauthors.com/eeo/promotion.

Thank you again for your contribution to Plant Direct. If you have any questions, feel free to contact the editorial office at plantdirect@wiley.com .

Sincerely,

Jyothilakshmi Vadassery

Jonathan Ingram

Editor, Plant Direct

-- Editor comments

-- Reviewer comments:

Reviewer #1:

The revised manuscript addressed the concerns raised.

Reviewer #2:

Thank you for your detailed response and the revisions made to address the comments. I appreciate the effort to clarify and expand the manuscript. However, several critical concerns remain unresolved, impacting the decision on acceptance.

• Overlap with Gutiérrez et al. (2014): While the novel findings of mitochondrial signaling and ANAC102's potential role in PCD modulation are noted, the core finding of RB-induced chloroplast-dependent PCD overlaps significantly with prior work. The novelty hinges on these new aspects, but their lack of robust validation limits their impact.

• Time Point for Transcriptomics (30 minutes): The justification for the 30-minute time point based on rapid response and viability drop (70% light-grown, 50% dark-grown at 2 hours, mostly dead at 24 hours) is acknowledged. However, the subjective nature of the viability argument and the disconnect from 24-hour PCD phenotypes weaken this choice. The proposed future RT-PCR time-series (e.g., 1h, 6h, 24h) with DREM analysis is a step forward, but its feasibility under current funding constraints raises concerns. Without interim data, the transcriptomic insights remain incomplete.

• ANAC102 Functional Validation: The identification of ANAC102 as a GRN hub is a promising finding, yet the absence of functional validation is a major gap. The reliance on literature support (Li et al., 2020; D'Alessandro et al., 2018) and the funding/expertise excuse do not suffice. A basic experiment, even if limited, would strengthen this claim significantly.

Response Letter 1:

Dear reviewers and editorial team,

we thank the reviewers and editor for their thoughtful and constructive feedback, and time spent on our manuscript. We particularly appreciate the recognition that presented results have important implications for understanding ROS-induced PCD and plant stress responses, including evidence for calcium and mitochondrial involvement in the Rose Bengal-induced cell death programme. We also confirmed that RB-induced PCD it is a light-dependent and chloroplast-driven death, and is associated with rapid transcriptional reprogramming (that does not occur in the dark, or in cells without chloroplasts), that we characterized using RNA-seq.

We have considered all the received comments carefully and modified the revised version of the

manuscript accordingly, as detailed below. Regarding the lack of functional validation, we acknowledge the reviewers' suggestion to functionally validate its role in RB-induced PCD. While our transcriptomic and network analyses identified ANAC102 as a potential transcriptional hub gene activated by RB treatment, it is important to clarify that this was just one of our findings and that the characterization of this gene was not the central focus of the study. Unfortunately, due to the conclusion of the funding period and the lack of available personnel with suitable expertise in the lab, we are not able to undertake additional experimental work currently.

Considering that the study highlighted the chloroplast dependency of RB-induced PCD, and complexity of signaling pathways involved in plant PCD, the functional validation of ANAC102's involvement would require setting up stable, photosynthetic cell suspension lines derived from ANAC102 overexpression and knockout plants. Considering broadly recognized role of ANAC102 in plant stress response, the high precision cell culture model would be in our view most appropriate in terms of providing quantitative, time-resolved insights into ANAC102 involvement in modulation of RB-induced cell death - but generating such cultures can take several months up to one year based on our experience. However, we have added references to more literature data that indirectly support the link between ANAC102 and singlet oxygen induced PCD to the revised manuscript, for example highlighting upregulation of this TF in crl mutant that shows localized cell death (Li et al., 2020), and the fact that anac102 mutants were sensitive to excessive light due to incomplete chloroplast retrograde signalling and showed increased leaf bleaching (D'Alessandro, Ksas & Havaux, 2018). We also we have revised the Discussion section to explicitly acknowledge lack of functional validation, emphasize the exploratory nature of the ANAC102 finding, and frame it as an observation that warrants future functional follow-up. We hope that the strength of the presented data, especially the mechanistic insights into singlet oxygen-triggered PCD via chloroplasts, calcium signaling, and mitochondrial participation, as well as bioinformatic analyses, along with our transparent discussion of limitations, supports the publication of the current manuscript as a valuable contribution to the field.

Collectively, we are thankful for the constructive review process that made the our manuscript

clearer, easier to follow, and ensured that it will be of interest to the broad plant science community.

Detailed responses to received comments:

Reviewer #1:

Jnaid and coworkers investigated the impact of Rose Bengal (RB), a photosensitizer as a precise pharmacological tool that can induce singlet oxygen activated and chloroplast-driven PCD in Arabidopsis thaliana cell suspension cultures in a light dependent manner. This PCD pathway is involves transcriptional reprogramming and requires calcium burst and mitochondrial assistance. Authors also showed that chemical inhibitors such as norflurazon, spectinomycin, or lincomycin inhibits this PCD but leads to necrosis. Calcium signalling inhibitor lanthanum chloride, and mitochondrial membrane protector cyclosporin A, also inhibits this PCD. Transcriptional changes within 30 minutes of RB treatment, revealed ANAC102 as a hub regulating a gene network driving this response.

Overall, the study leads to an understanding of oxidative stress response in cell suspension cultures, which may find applications in enhancing plant resilience to environmental stressors. However, the impact on live plants could have been much better to deduce such conclusions.

➢ We thank the reviewer for their kind comments.

Specific comments:

1. The study could have been extrapolated in live plants as RB treatment is always a challenge in live plants compared to individual cells.

➢ Regarding lack of experimentation of whole plants, the use of Arabidopsis suspension cell cultures in this study provided a highly controlled and responsive model for dissecting the chloroplast-dependent PCD triggered by RB. This system allowed for direct and quantitative comparison between chloroplast-containing and chloroplastdepleted cells, which would be hard to achieve using whole plants. Moreover, the homogeneity of cell populations, their feasibility to pharmacological manipulations and ease of microscopic examination enabled precise, time-resolved characterization of cell death dynamics, distinguishing between regulated and uncontrolled cell death pathways and quantitatively determinating the rates of both. However, we recognize that while this

model system allows to establish core mechanistic insights, future work in whole plants will be necessary to validate these findings in the context of plant physiological responses at the organismal level. We have included this comment in the conclusions section of revised manuscript.

2. Most if these findings are well established in terms of singlet oxygen triggered and chloroplast driven PCD, Please refer to work done on flu, chlorina 1 and crumpled leaf mutants under dark-to-light transitions and high light stresses
(relevant publications https://doi.org/10.1105/tpc.112.100479 already
, https://doi.org/10.1105/tpc.18.00578 done, https://doi.org/10.1105/tpc.20.00016)

➢ Thank you for this suggestion. Excellent idea - we have included references to PCD phenotypes in singlet oxygen mutants in the discussion as recommended.

3. ANAC102 is known to regulate a network of genes involved in xenobiotic responses mainly detoxification and not PCD (work on chlorina 1and crumpled leaf mutants; https://doi.org/10.1105/tpc.18.00578, https://doi.org/10.1105/tpc.20.00016). It's precise role in PCD is not established, it could have been tested using mutant and overexpression lines under RB treatments.

➢ As outlined in the start of this letter, we definitely agree that the role of ANAC102 in PCD modulation remains to be fully elucidated. Our study does not claim to definitively establish its function in RB-induced PCD, but rather highlights ANAC102 as a TF of interest in this context based on network analysis and its known involvement in stress responses. Regarding functional validation, we acknowledge in the discussion that testing levels of RB-induced PCD in mutant or overexpression lines would be the next step to test ANAC102's involvement. However, due to the conclusion of the funding period and the absence of available personnel with the required expertise, we are currently unable to undertake these experiments. Nonetheless, we believe that our identification of ANAC102 within the RB-responsive GRN, especially its connection to known singlet oxygen and chloroplast retrograde signaling, makes it a valuable candidate for future functional studies that is worth highlighting in the manuscript. We have added references to more literature data that indirectly support the link between ANAC102 and singlet

oxygen induced PCD to the revised manuscript, for example highlighting upregulation of this TF in crl mutant that shows localized cell death (Li et al., 2020), and the fact that anac102 mutants were sensitive to excessive light due to incomplete chloroplast retrograde signalling and showed increased leaf bleaching (D'Alessandro, Ksas & Havaux, 2018)

Reviewer #2:

The manuscript presents a compelling and well-structured study investigating the use of Rose Bengal (RB) as a photosensitizer to induce chloroplast-driven programmed cell death (PCD) in Arabidopsis thaliana cell suspension cultures. The findings have important implications for understanding ROS-induced PCD and plant stress responses. However, there are several areas that require clarification and improvement before the manuscript can be considered for publication.

➢ We thank the reviewer for their kind words and useful suggestions on how to improve the manuscript.

Introduction:

• The rationale for using Arabidopsis cell suspension cultures is not clearly explained. Including a brief justification (e.g., suitability for microscopy, uniform responses, ease of chemical treatment) would help non-expert readers understand the advantages of this model system.

➢ This is a great point. We have expanded this in the introduction.

• The Introduction focuses heavily on singlet oxygen ($^1O_2$) and chloroplast-derived ROS, while other well-established PCD triggers (e.g., developmental cues, pathogen response, abiotic stress) are underrepresented. A more balanced overview would improve the context of the study.

• A brief overview of the general concept of PCD in plants would enhance clarity, particularly for a broader readership.

➢ We have slightly expanded the sections of introduction that provide general introduction of PCD in plants in context of development, pathogen response and abiotic stress in the revised manuscript, while being mindful of avoiding making the introduction excessively long. We think that improved the manuscript – thank you!

Potential limitations of RB, such as its light-independent toxicity, are not acknowledged in the

Introduction. This should be briefly mentioned to present a balanced view from the outset.

➢ We acknowledge the dark independent toxicity already both in the abstract (line 28) and in the introduction (line 133).

The roles of mitochondria and calcium signaling in PCD are central to the study's findings but are not adequately introduced. Including a few sentences about their relevance would better connect the Introduction to the Results.

➢ Good point, we added extra information on chloroplast and mitochondira in context of PCD with appropriate reference.

Results:

• The results convincingly show that RB induces light- and chloroplast-dependent PCD, with higher PCD rates observed at 0.5 µM compared to 0.1 µM. This is well supported by the use of chloroplast inhibitors (norflurazon, spectinomycin, lincomycin) and chlorophyll measurements (Fig. 1A-F).

• A novel and interesting finding is the light-independent (dark) toxicity of RB, particularly in dark-grown cells, which leads to necrosis. This adds originality to the study.

• Calcium and mitochondrial signaling are thoroughly investigated using pharmacological inhibitors ($LaCl_3$, CsA, and 3-MA), and the time-course experiments are well designed, aligning with known PCD pathways.

• The transcriptomic analysis reveals 187 differentially expressed genes (DEGs) associated with stress response and metabolic shutdown. The gene regulatory network (GRN) highlights ANAC102 as a potential key regulator.

➢ Thank you for your positive comments on the novelty of the work carried out.

Weaknesses and Suggestions for Improvement:

1. Dark Toxicity Mechanism:

The mechanism behind RB's light-independent dark toxicity is not explored in depth. Further investigation or at least a discussion of possible pathways would improve the understanding of RB's non-specific effects.

➢ We agree – it would be great to identify the mechanism underlying the dark toxicity of RB. We expanded discussion of this in the revised manuscript.

2. Limited RB Concentration Range:

Only two concentrations of RB (0.1 µM and 0.5 µM) were tested. Including additional concentrations would strengthen the analysis of dose-dependent effects. The authors should consider expanding this range or providing justification for the chosen doses.

➢ This is a good point, we haven't explained this well enough, this is now amended in the revised manuscript in results section. We selected 0.5 uM RB based on the previous study on PCD induced by short term exposure to RB (Gutiérrez et al 2014) and decided to include a lower concentration to check for dose-dependent effects and because we studied the effect of prolonged exposure.

3. Single-Time-Point Transcriptomics:

RNA-seq was conducted at a single time point (30 minutes post-treatment), which likely captures only early transcriptional changes. The manuscript should clarify the reasoning behind this time point selection. Additionally, it is not clear how many independent biological replicates were included in the RNA-seq experiment-this should be explicitly stated to evaluate data robustness. To gain further insight into temporal gene regulation, it is recommended to perform RT-qPCR validation of ANAC102 and other key GRN genes at additional time points (e.g., 1 h, 6 h, 12 h, and 24 h).

➢ Good point. There were two key reasons for selection of this early time point for the RNA-seq to study the response of light and dark grown cells, treated with RB under light and dark conditions (8 different treatment-condition combinations, three independent repeats of experiment carried out – the level of replication has now been clarified in the revised manuscript).

Firstly, Rose Bengal when exposed to light, can generate singlet oxygen, that is a highly reactive, short-lived intermediate, therefore changes in gene expression that drive decisions for cell death and survival are likely to be rapid. Further, based on our time course analyses included in the manuscript, harvesting RNA from cells at e.g. 2 hrs of treatment with RB would mean testing gene expression in the samples where only ~70% (light grown) and ~50% (dark grown) cells remain alive, with the viability dropping further at subsequent time points with vast majority of cells dead at 24hr. Based on this

we think that capturing transcriptional response and construction of GRN underlying the signaling to RB is most likely at the early time point we used. Secondly, the previous study on RB-induced PCD used microarrays to study transcriptional changes was carried out at this time point (for light grown cells in the light only), and use of the same time point facilitated comparisons between studies.

At this stage, we are constrained by lack of funding and personnel (the project has been completed) and unable to carry out further experimental work. However, we highlight the need of multi-time point transcriptome profiling of RB-induced changes in gene expression in the discussion – this albeit more costly than qPCR of several genes from the 30 min early response GRN at more time points – could allow using computational tools such as DREM (Dynamic Regulatory Events Miner) to identify dynamic transcriptional regulators and bifurcation points in gene regulatory networks underlying responses to RB.

4. Lack of Functional Validation for ANAC102:

While ANAC102 is highlighted as a key node in the GRN, the manuscript lacks any experimental validation. Even preliminary functional assays would significantly strengthen the claim of ANAC102's regulatory role in RB-induced PCD.

➢ As outlined in the start of this letter, we agree that the role of ANAC102 in PCD modulation remains to be fully elucidated. Our study does not claim to definitively establish its function in RB-induced PCD, but rather highlights ANAC102 as a TF of interest in this context based on network analysis and its known involvement in stress responses. Regarding functional validation, we acknowledge in the discussion that testing levels of RB-induced PCD in mutant or overexpression lines would be the next step to test ANAC102's involvement. However, due to the conclusion of the funding period and the absence of available personnel with the required expertise, we are currently unable to undertake these experiments. Nonetheless, we believe that our identification of ANAC102 within the RB-responsive GRN, especially its connection to known singlet oxygen and chloroplast retrograde signaling, makes it a valuable candidate for future functional studies that is worth highlighting in the manuscript. We have added references

to more literature data that indirectly support the link between ANAC102 and singlet oxygen induced PCD to the revised manuscript, for example highlighting upregulation of this TF in crl mutant that shows localized cell death (Li et al., 2020), and the fact that anac102 mutants were sensitive to excessive light due to incomplete chloroplast retrograde signalling and showed increased leaf bleaching (D'Alessandro, Ksas & Havaux, 2018)
